# Screening Tool for Paroxysmal Atrial Fibrillation Based on a Deep-Learning Algorithm Using Printed 12-Lead Electrocardiographic Records during Sinus Rhythm

**DOI:** 10.31083/j.rcm2507242

**Published:** 2024-07-02

**Authors:** Yang Zhou, Deyun Zhang, Yu Chen, Shijia Geng, Guodong Wei, Ying Tian, Liang Shi, Yanjiang Wang, Shenda Hong, Xingpeng Liu

**Affiliations:** ^1^Heart Center, Beijing Chaoyang Hospital, Capital Medical University, 100020 Beijing, China; ^2^HeartVoice Medical Technology, 230027 Hefei, Anhui, China; ^3^Department of Cardiology, Peking University International Hospital, 100094 Beijing, China; ^4^National Institute of Health Data Science, Peking University, 100191 Beijing, China

**Keywords:** artificial intelligence, screening tool, paroxysmal atrial fibrillation, printed electrocardiography, sinus rhythm

## Abstract

**Background:**

Recent advancements in artificial intelligence (AI) have significantly improved atrial fibrillation (AF) detection using electrocardiography (ECG) data obtained during sinus rhythm (SR). However, the utility of printed ECG (pECG) records for AF detection, particularly in developing countries, remains unexplored. This study aims to assess the efficacy of an AI-based screening tool for paroxysmal AF (PAF) using pECGs during SR.

**Methods:**

We analyzed 5688 printed 12-lead SR-ECG records from 2192 patients admitted to Beijing Chaoyang Hospital between May 2011 to August 2022. All patients underwent catheter ablation for PAF (AF group) or other electrophysiological procedures (non-AF group). We developed a deep learning model to detect PAF from these printed SR-ECGs. The 2192 patients were randomly assigned to training (1972, 57.3% with PAF), validation (108, 57.4% with PAF), and test datasets (112, 57.1% with PAF). We developed an applet to digitize the printed ECG data and display the results within a few seconds. Our evaluation focused on sensitivity, specificity, accuracy, F1 score, the area under the receiver-operating characteristic curve (AUROC), and precision-recall curves (PRAUC).

**Results:**

The PAF detection algorithm demonstrated strong performance: sensitivity 87.5%, specificity 66.7%, accuracy 78.6%, F1 score 0.824, AUROC 0.871 and PRAUC 0.914. A gradient-weighted class activation map (Grad-CAM) revealed the model’s tailored focus on different ECG areas for personalized PAF detection.

**Conclusions:**

The deep-learning analysis of printed SR-ECG records shows high accuracy in PAF detection, suggesting its potential as a reliable screening tool in real-world clinical practice.

## 1. Introduction

Atrial fibrillation (AF) is a common arrhythmia associated with an elevated risk of stroke, heart failure, and cardiovascular mortality [1, 2, 3]. Unfortunately, AF often goes unnoticed and untreated due to its frequent asymptomatic or minimally symptomatic nature, especially in cases of paroxysmal AF (PAF) [4]. In China, over one-third of AF patients remain unaware of their condition, with a higher prevalence observed in specific demographics such as individuals aged 45–54 years and ≥75 years, men, rural residents, those with lower education, and patients without major vascular comorbidities [5]. Thus, strategies for screening and identifying undetected AF are of great importance in stroke prevention.

Screening for AF using just a standard 12-lead electrocardiography (ECG) is challenging. While continuous monitoring using cardiac implantable electronic devices (e.g., pacemaker, implantable defibrillator, implantable loop recorder) has improved AF detection rates [6, 7], these methods have provided few benefits and contributed to increased healthcare costs. Multiple wearable devices such as smartwatches, smartphones, and digital ECG patches have been proposed as tools for AF screening. However, to date, these screening modalities are neither practical nor affordable [8, 9, 10, 11].

While artificial intelligence-enabled electrocardiography (AI-ECG) has recently gained attention as a potent and cost-effective tool for detecting or predicting PAF during sinus rhythm (SR), no prior study has explored the potential of conventional printed ECG (pECG) as a screening tool for PAF. The use of AI-pECG has the potential to bridge a clinical gap, especially in developing countries like China. In this study, our objectives were two-fold: (1) to assess the suitability and accuracy of a deep-learning model designed to detect AF using printed ECG records, and (2) to develop a deep learning-based WeChat applet for AF screening that can provide immediate results in real-world clinical settings.

## 2. Methods

### 2.1 Study Population and Data Collection

This single-center, retrospective study included patients aged 18 years or older who underwent catheter ablation for PAF and other electrophysiological procedures at Beijing Chaoyang Hospital between May 2011 and August 2022. Printed ECG records from at least one 12-lead SR-ECG (25 mm/s, 10 mm/mV) obtained upon admission were included. ECG data and demographic characteristics were extracted from the database. When more than one eligible ECG was found, we used all ECGs. Exclusions included ECG records with poor-quality tracing or non-standard typesetting (12‑by‑1), as illustrated in the **Supplementary Fig. 1**. Printed ECG records were digitized into images using a Canon CanoScan LiDE 300 scanner (Manufacturer: Canon Inc., Location: Tokyo, Japan) and stored in JPG format. Diagnostic labels were assigned by an electrophysiologist: patients with at least one recorded AF rhythm were categorized into the AF group, while those with no AF-ECG records and no reference to AF in their medical history and electrophysiological examinations were assigned to the non-AF group. The Beijing Chaoyang Hospital Institutional Research Board approved the study protocol and all ethical considerations. The need for informed consent was waived by the ethics board of our hospital because the images had been acquired during daily clinical practice.

### 2.2 Data Processing, Model and WeChat Applet Development

Before training, we adjusted the angles of the printed ECG images to ensure that all ECG waveforms were displayed in a horizontal position, and we deleted the identifying information of all patients. Deep neural networks were built by using the *EfficientNet-V2 Network * [12], a multidimensional mixed model scaling method, to integrate all scanned ECG images for the detection of AF. The model was then pre-trained on the ImageNet data set to learn the common semantic representations in classification tasks, and the pre-trained model was subsequently applied to the target task. The *np.random.shuffle* (https://numpy.org/) function was used to shuffle the order of the patients, who were then assigned to the training set, validation set, and test set with a ratio of 9:0.5:0.5. Both types of diagnostic labels (AF and non-AF) were equally represented within each dataset. Subsequently, the WeChat applet was developed based on the AI-pECG model. The applet could be accessed by scanning the quick response (QR) code (**Supplementary Fig. 2**). ECG images were input into the WeChat applet, which automatically displayed the diagnostic label of AF or non-AF.

### 2.3 Visualization of Results

To enhance our understanding of the model and facilitate further comparison with existing methods, we aimed to identify the sections of the pECGs that played a significant role in the detection of PAF within this algorithm. We employed a gradient-class activation map (Grad-CAM) as a sensitivity map, utilizing the gradient information of the algorithm for visualization, then highlighted the ECG regions that were relevant to our endpoint [13]. If the probability of a classifier was sensitive to a specific region of the signal, the region would be considered significant in the model.

### 2.4 Statistical Analysis

Categorical data are presented as frequencies and percentages, with the chi-square (χ^2^) test comparing the distribution of these categorical variables between groups. Continuous variables with a normal distribution are expressed as mean ± standard deviation (*x ± s*), while those with a non-normal distribution were presented as median (Q1, Q3). The independent sample *t*-test was used to compare the variables between the groups, and the IBM SPSS version 26.0 (IBM Corp., Armonk, NY, USA) was employed for data analysis.

To assess the performance of the model in detecting PAF based on pECG data, we calculated the following key metrics: sensitivity, specificity, accuracy, F1 score, the area under the receiver-operating characteristic curve (AUROC), and the area under precision-recall curves (PRACU). The PR and ROC curves were generated using Python 3.7 (https://www.python.org/), Matplotlib 3.0.2 (https://matplotlib.org/stable/plot_types/basic/bar.html) and Scikit-learn 1.2.1 (https://scikit-learn.org/stable/index.html).

## 3. Results

### 3.1 Baseline Characteristics

**Fig. 1.**
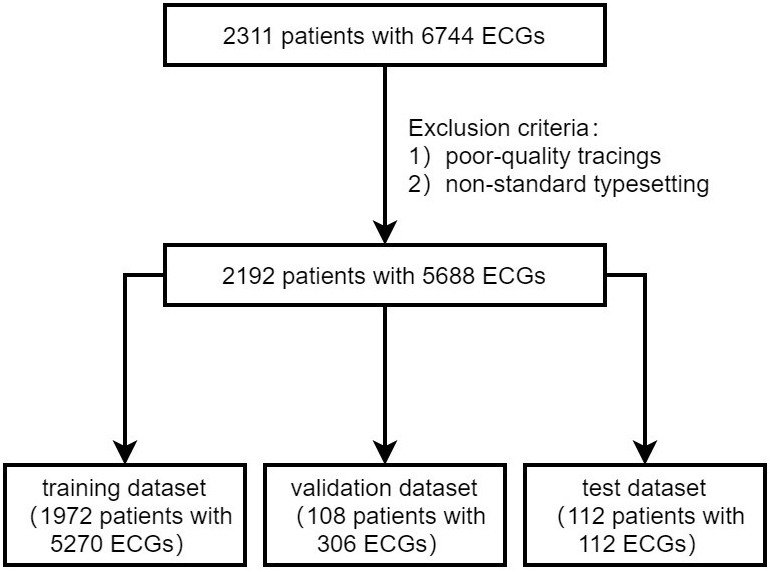
**Patient flow diagram.** ECG, electrocardiography.

In our analysis, we included data from 2311 patients, which comprised 6744 SR-ECGs, as illustrated in Fig. 1. Following the application of exclusion criteria (n = 119), we focused on 2192 cases with 5688 SR-ECGs upon admission for further analysis. Among them, there were 1255 (57.3%) PAF patients. These cases were randomly distributed across three datasets, with 1972 (57.3% with PAF), 108 (57.4% with PAF), and 112 (57.1% with PAF) cases, respectively. The training and validation sets included 5576 standard 12-lead ECGs with a median of one ECG per individual (interquartile range [IQR]: 1–3), and the test set comprised 112 ECGs (one ECG per individual). The baseline clinical characteristics of the patients are shown in Table 1. Participants had a mean age of 62.8 years (standard deviation, SD 13.9) and 1213 (55.3%) of them were women.

**Table 1.** **Patient clinical characteristics**.

Characteristics	All patients	PAF	Non-PAF
	(n = 2192)	(n = 1255)	(n = 937)
Age, y	62.8 ± 13.9	63.4 ± 10.7	61 ± 17.2
Female sex	1213 (55.3%)	721 (57.5%)	492 (52.5%)
BMI (${\mathrm{kg/m}}^{2}$)	24.7 ± 4.0	25.8 ± 3.7	24.7 ± 2.0
Smoke	448 (20.4%)	320 (25.5%)	128 (13.7%)
Alcohol	475 (21.7%)	286 (22.8%)	189 (20.2%)
Hypertension	1144 (52.2%)	769 (61.3%)	375 (40.0%)
Diabetes mellitus	344 (15.7%)	185 (14.7%)	159 (17.0%)
Coronary artery disease	289 (13.2%)	136 (10.8%)	153 (16.3%)
Stroke	119 (5.4%)	51 (4.1%)	68 (7.3%)
Vascular disease	533 (24.3%)	314 (25.0%)	219 (23.4%)
Hyperthyroidism	23 (1.0%)	9 (0.7%)	14 (1.5%)
COPD	19 (0.9%)	12 (1.0%)	7 (0.7%)
Heart failure	196 (8.9%)	130 (10.4%)	66 (7.0%)
CHA2DS2-VASc score	2.3 ± 1.8	2.0 ± 1.6	2.6 ± 2.2

y, years; PAF, paroxysmal atrial fibrillation; BMI, body mass index; COPD, chronic obstructive pulmonary disease; CHA2DS2-VASc score calculated as congestive heart failure, hypertension, age 75 years and older, diabetes, stroke or transient ischemic attack, vascular disease, age 65 to 74 years, and sex category.

### 3.2 AI Algorithm Performance

**Fig. 2.**
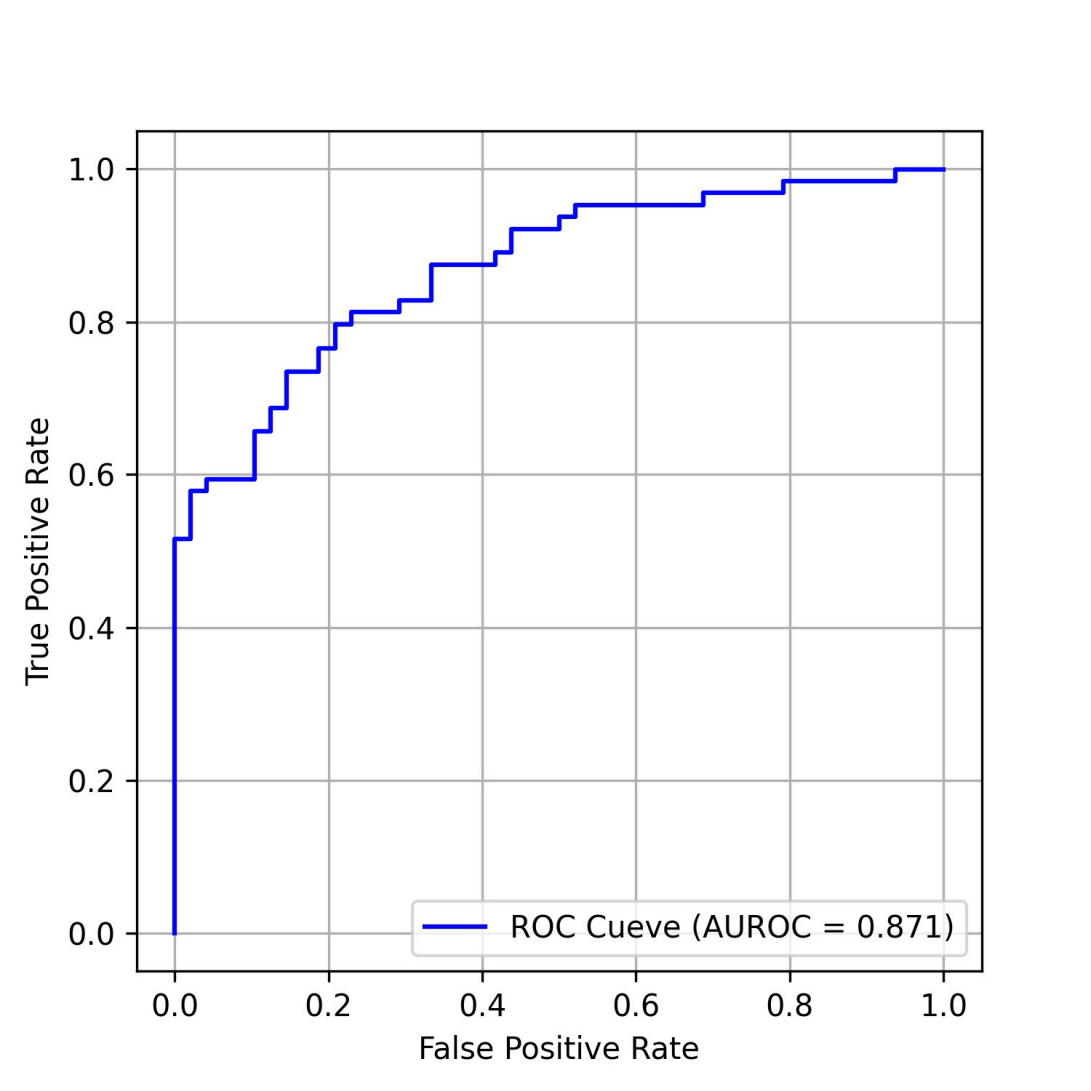
**ROC curves of the model on the test data set. **AUROC, area under the receiver operating characteristic curve; ROC, receiver operating characteristic curve.

**Fig. 3.**
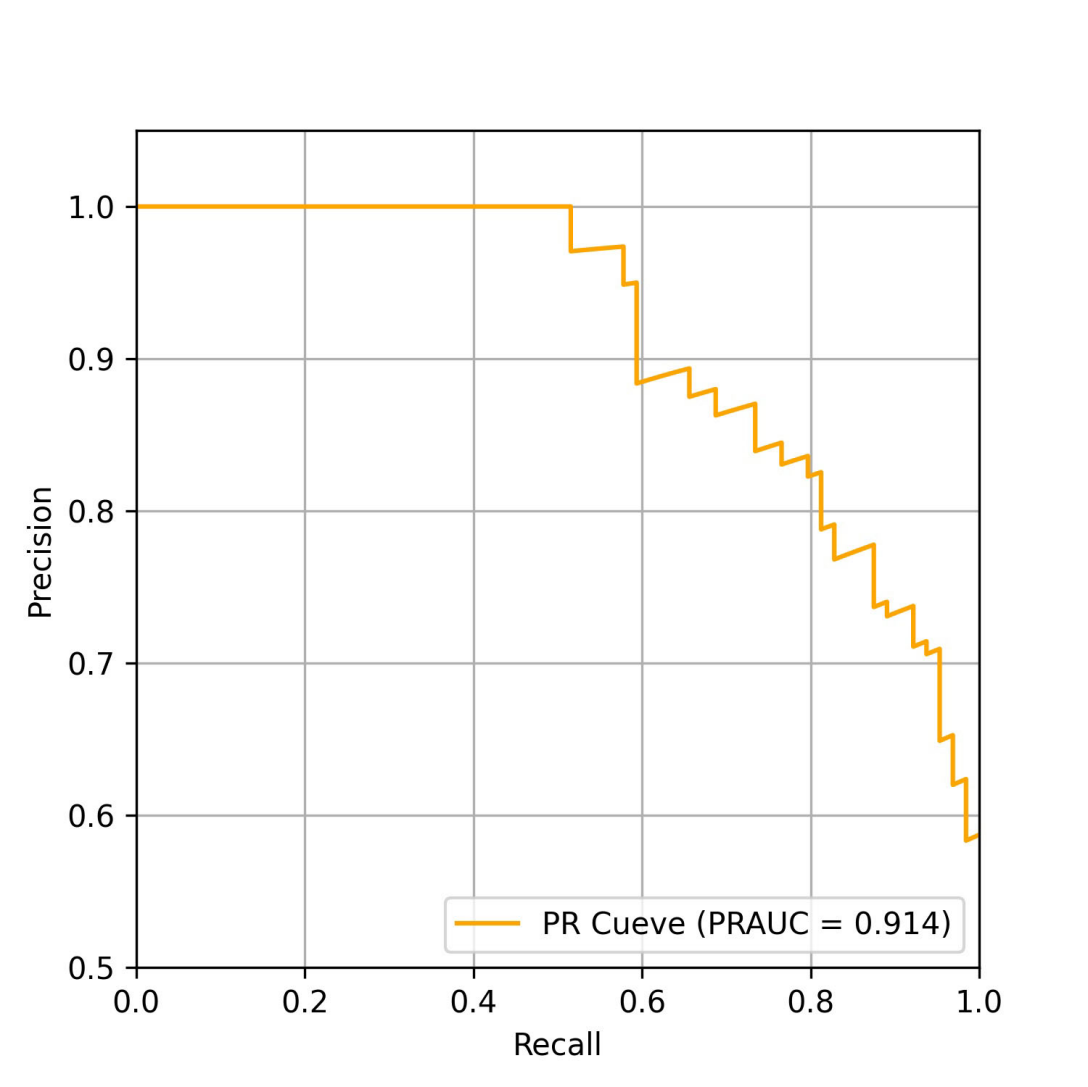
**Precision-recall curve of the model on the test data set.** PRAUC, area under the precision-recall curve; PR, precision recall.

The performance of the AI-pECG model in detecting PAF was notable. The model achieved an area under the receiver-operating characteristic curve (AUROC) of 0.871, as depicted in Fig. 2, along with an area under the precision-recall curve (PRAUC) of 0.914, illustrated in Fig. 3. The model demonstrated a sensitivity of 87.5%, specificity of 66.7%, accuracy of 78.6%, and an F1 score of 0.824. The optimal sensitivity threshold for accurate disease classification was determined to be 0.416. While acknowledging a slight increase in the misdiagnosis rate, it is crucial to highlight that the model significantly reduces the probability of missed diagnoses, showcasing its potential clinical applicability.

### 3.3 ECG Regions of Interest Identified by the AI Model for AF Detection Using Attention Techniques

**Fig. 4.**
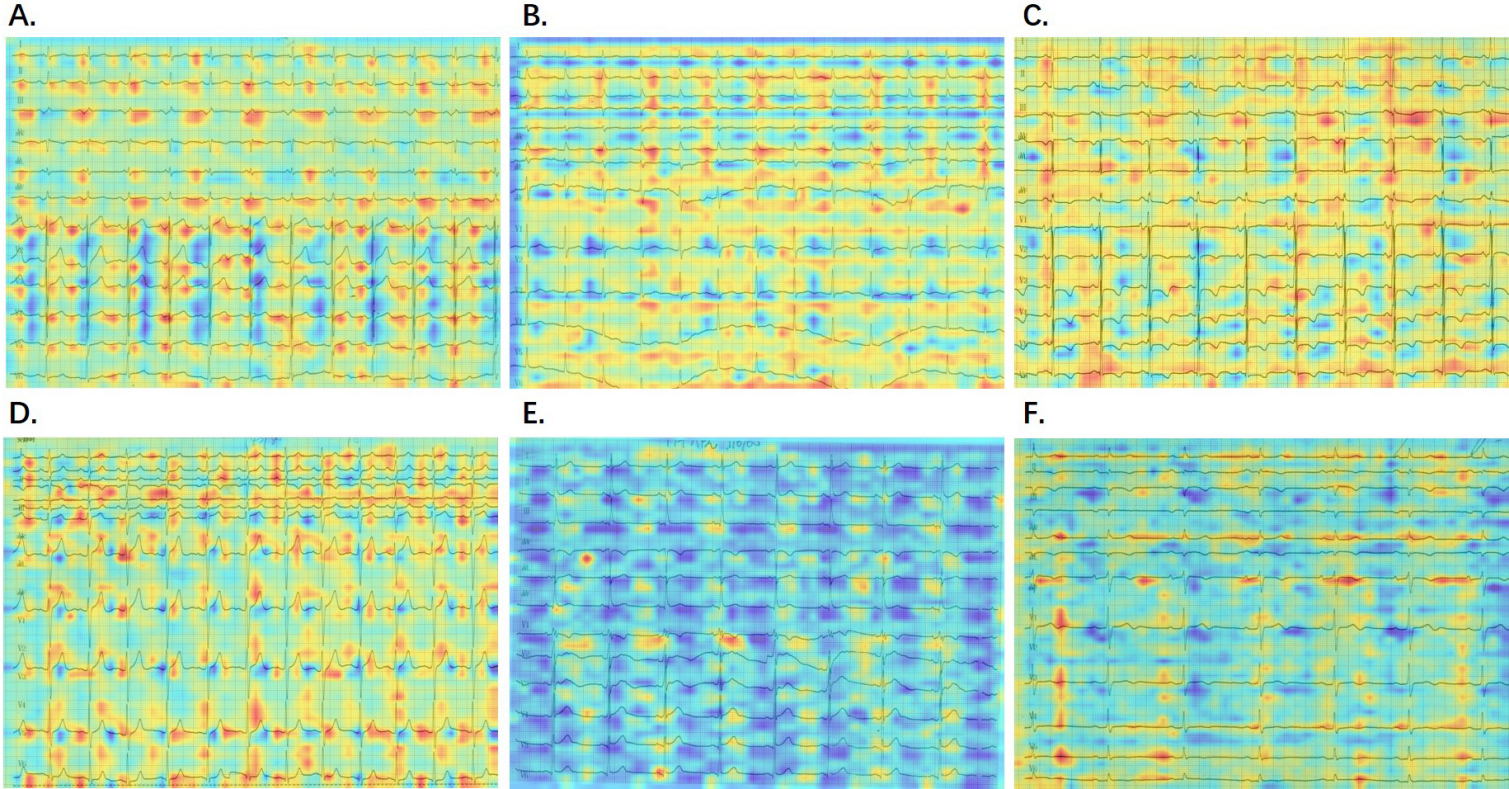
**Visualization of AI model detection.** (A–C) AF group with corresponding probabilities of 0.9925, 0.9167, 0.7719. (D–F) Non-AF group with corresponding probabilities of 0.4035, 0.3809, 0.3670. Red areas indicate regions influencing a positive detection, while blue areas represent regions influencing a negative detection. AI, artificial intelligence; AF, atrial fibrillation.

To identify the specific regions the AI model concentrated on when detecting PAF from printed SR-ECGs, we employed a gradient-class activation map (Grad-CAM). This technique highlighted the non-trainable focus areas of a convolutional neural network (CNN)-based model, calculated through the model’s internal gradient and feature map output. This enabled the identification of focal points prioritized by the model. A total of 224 sensitivity maps (112 in each group) were randomly selected from the entire dataset and confirmed through Grad-CAM. As depicted in Fig. 4, the red regions display a degree of instability across various images.

### 3.4 Procedures for the Screening Tool based on the AI-pECG Model

**Fig. 5.**
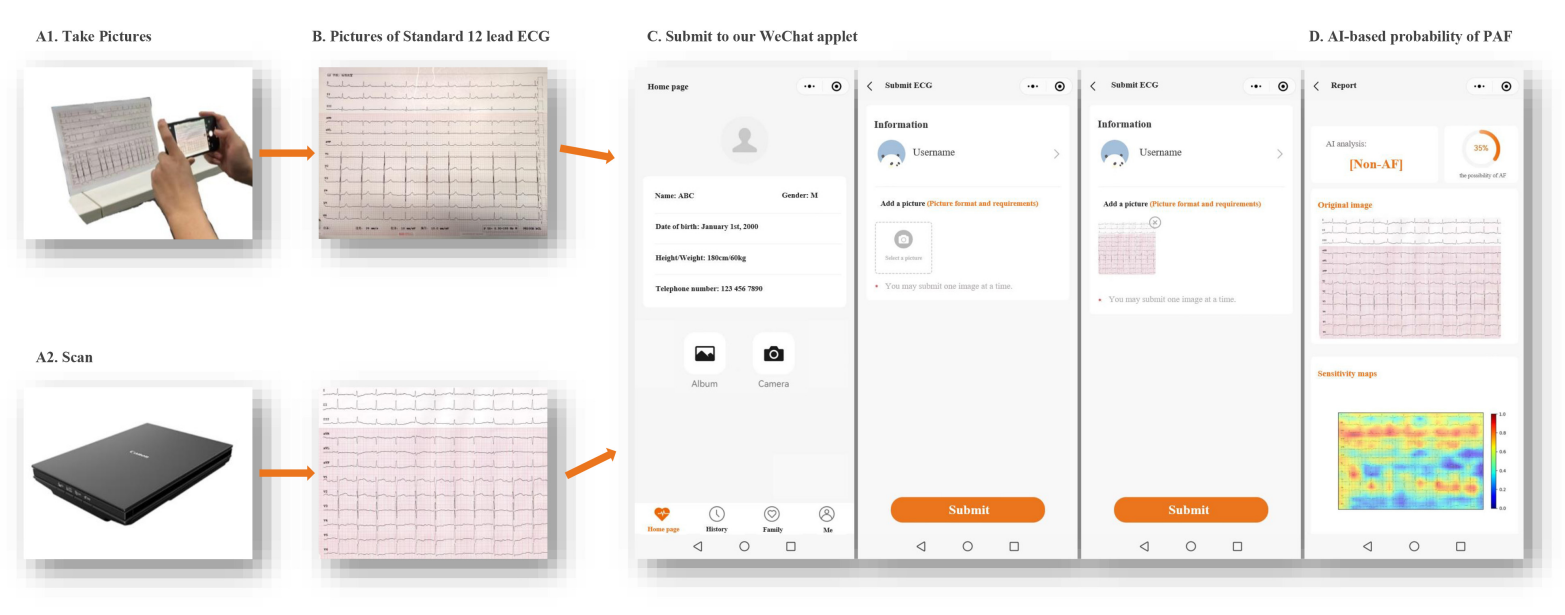
**Flow diagram of screening for PAF using the WeChat applet based on AI and printed ECG during sinus rhythm. **PAF, paroxysmal atrial fibrillation; AI, artificial intelligence; ECG, electrocardiography. (Manufacture: Canon Inc. https://canon.jp/)

Our model is designed to capture or scan paper ECG images using a mobile phone camera or a scanner. Subsequently, by scanning the WeChat QR code and entering our applet, they can upload the image. The process is swift and user-friendly, with results generated in seconds. This is guided by clear step-by-step prompts (Fig. 5).

## 4. Discussion

In this study, we presented a novel deep-learning algorithm designed for the identification of PAF based on printed 12-lead ECG records obtained during SR. Our innovative deep-learning model, coupled with a companion WeChat applet, provides a practical solution for clinicians, particularly those in developing countries, to conduct real-time AF screening with an impressive AUROC of 0.871 and high sensitivity (87.5%). The study also highlights features of ECG that are critical to identifying PAF. While these features may be challenging to visualize, they are identifiable through the AI algorithm. To the best of our knowledge, this study is the first attempt to integrate pECG records and AI into a model for AF detection. The mobile user interface has proven to be intuitive, user-friendly, and compatible with a range of widely-used devices.

Early diagnosis of AF, particularly PAF, is clinically beneficial but presents significant challenges. Approximately one-third of AF patients are asymptomatic, and the sporadic nature of symptoms hinders precise ECG monitoring during AF episodes [14]. Previous studies focused on P-wave indices, such as duration, dispersion, area, abnormal axis, and P-wave terminal force in lead V1 during SR, as predictors for the development of AF [15, 16, 17, 18]. Advanced interatrial block has also been identified as a significant predictor of new-onset AF, AF recurrence, and ischemic stroke [19]. The independent predictive value of QRS duration for new-onset AF in women was highlighted by Aeschbacher *et al*. [20]. The QRS duration reflects the conduction speed through the specialized cardiac conduction system and ventricular myocardium [21]. It was demonstrated that this variable is intricately linked to cardiac structural and functional abnormalities [21]. However, existing methods have shown moderate performance in providing clinical utility. Standard SR-ECG readings do not always represent normal atrial contraction uniformly [22]. A substantial proportion of patients with post-cardioversion AF continue to exhibit persistent disorganized appendage contraction [22]. This suggests that there may be multiple wavelets, undetectable to the human eye, that precede the typical ECG waveform changes in individuals predisposed to AF. These factors collectively underscore the complexity of early PAF detection and the need for more refined diagnostic methods.

Recent studies have emphasized the potential of AI algorithms to address these challenges in AF detection and prediction. Attia *et al*. [23, 24] developed an automatic AF detection method using a CNN model based on short-term normal ECG signals, indicating that a shorter period (<30 days) between AF and SR enhances diagnostic accuracy. In a follow-up study, they analyzed surface ECGs from a substantial cohort comprising 59,212 patients, both before and after the first documented AF episode. This study revealed the AI-ECG probability of AF gradually increases prior to the first AF episode, experiences a temporary decrease 1–2 years post-AF, and then resumes a continuous increase thereafter. Baek *et al*. [25] reported that an AI model showed that the optimal interval for detecting subtle changes in PAF occurred within 0.24 seconds before the QRS complex in the 12‑lead ECG during SR. This insight contributes to the evolving landscape of AI-based predictive tools, which estimate the probability of developing AF from surface ECGs recorded during sinus rhythm, showing significant promise in clinical practice.

All previous AI-ECG studies utilized digital ECG data, which is not universally available in developing countries [26, 27]. The majority of hospitals in developing countries do not yet benefit from the digital recording and storage of ECG data, and do not have the ability to automatically reanalyze paper-based ECG records. The collection of extensive, high-quality ECG data remains a challenge in these settings. However, deciphering the patterns that pECG records may reveal is an essential step toward developing a more accurate method for detecting PAF in low-resource clinical scenarios.

In our study, the AI model enabled the identification of subtle yet crucial differences between the pECGs obtained during SR from patients with PAF and those obtained from participants without a history of AF, even with relatively limited data. Although our model achieved a slightly lower ROC and accuracy compared with the models used in other recently published studies, our findings remain promising for clinicians treating patients in low-resource environments.

The preliminary screening tool could be used in the following manner: if the result indicates the presence of PAF, healthcare professionals should recommend more intensive monitoring for the patient. Conversely, for patients identified as non-AF, the routine AF education and observation would be recommended. This strategy could expedite the time to PAF diagnosis, thereby contributing to the prevention of ischemic stroke. Additionally, it may mitigate unnecessary medical interventions and decreasing the risk of bleeding associated with false-positive results during initial assessments. While it is premature to make any changes in the current AF recommendations, our findings represent a significant step forward in the field of AF screening.

The perception of AI algorithms as “black boxes” due to their opaque decision-making processes is a notable concern. In medical settings understanding the rationale behind diagnostic or therapeutic recommendations is essential for building trust and ensuring patient compliance [28]. This lack of transparency can be a significant barrier to the integration of AI systems in healthcare. To address this issue, researchers have investigated attention mechanisms such as Grad-CAM [29, 30, 31]. Kim *et al*. [32] observed a consistent focus in their model’s attention during predictions across window periods of 1, 2, and 4 weeks. The model’s identified focal points, specifically around one QRS point and one point between the T and P waves, provide valuable insights for exploring the mechanisms of AF through AI-ECG analysis.

In this study, we observed that in specific images, the highlighted red regions (indicative of the model’s focus) may concentrate on specific waveforms or electrocardiographic areas. Interestingly, these focal points might shift or appear in different positions in other images. This variability could indicate the model’s sensitivity to individual differences in diverse samples or stem from subtle uncontrollable variations in light and color among numerous print-scanned data. We will undertake further in-depth analyses of printed data to enhance result accuracy and clarity.

A few important limitations of the present study warrant discussion. First, our dataset, obtained from a single tertiary care teaching hospital in China, is relatively smaller in comparison to other large-scale AI-ECG studies. In order to ensure a robust sample size for model training in both groups, we designed the cohort with a higher prevalence of AF. Ongoing efforts are directed towards a prospective multicenter study aimed at evaluating the model’s performance across a more diverse, ostensibly healthy population. Second is the potential for undetected AF in patients categorized as non-AF, as our inclusion criteria were restricted to those who underwent electrophysiological procedures. It is reasonable to hypothesize that some false-positive patients in the non-AF group may be undiagnosed AF cases. Third, the image quality check process is current undergoing refinement, which limits the effectiveness of our WeChat applet. Finally, all pECGs recorded in our study were in a 12-by-1 ECG format, raising uncertainty about the generalizability of our results to other distributions. Our future studies aim to further explore the intricacies of pECGs to advance their clinical utility in precision medicine.

## 5. Conclusions

Utilizing deep-learning for the analysis of 12-lead printed ECG records has demonstrated its capability to accurately detect PAF during SR. This technology holds promise as a reliable screening tool in real-world clinical settings, particularly in regions with limited access to digital ECG analysis. The ongoing exploration of printed ECG patterns aims to unlock additional insights for precision medicine and clinical practice.

## Data Availability

The data underlying this article will be available upon reasonable request to the corresponding author.

## Abbreviations

AF, atrial fibrillation; ECG, electrocardiography; SR, sinus rhythm; pECG, printed electrocardiography; PAF, paroxysmal atrial fibrillation; AUROC, area under the receiver operating curve; PRAUC, area under the precision-recall curve; AI-ECG, artificial intelligence-enabled electrocardiography; Grad-CAM, gradient-weighted class activation map; CNN, convolutional neural network.

## Supplementary Material

Supplementary material associated with this article can be found, in the online version, at https://doi.org/10.31083/j.rcm2507242.
