# Supplementary material for: Screening Tool for Paroxysmal Atrial Fibrillation Based on a Deep-Learning Algorithm Using Printed 12-Lead Electrocardiographic Records during Sinus Rhythm
Source: Rev Cardiovasc Med. 2024 Jul 2;25(7):242. doi: 10.31083/j.rcm2507242 (PMC11317324; doi:10.31083/j.rcm2507242)
Supplement: Supplementary file 1 [file 2153-8174-25-7-242-s1.pdf]

# Supplemental material

**Supplementary Fig. 1. Standard ECG typesetting (12*1) for this study**

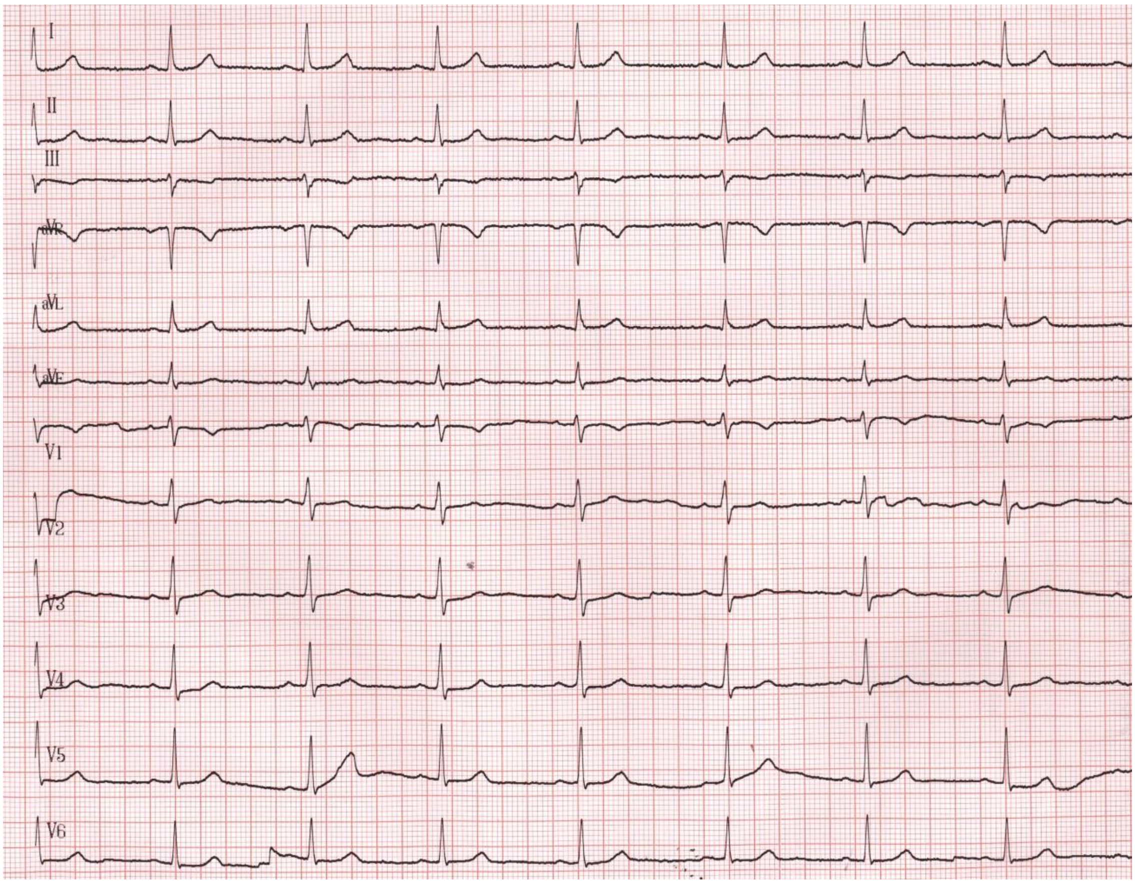

**Supplementary Fig. 2. QR code of the WeChat applet for detecting AF model**
